# Supplementary material for: Polyketide synthase gene clusters in the ericoid mycorrhizal fungus Oidiodendron maius: genome-wide prediction, expression profiling, and functional characterization of knockout mutants
Source: Front Plant Sci. 2026 Sep 16;17:1924430. doi: 10.3389/fpls.2026.1924430 (PMC13623903; doi:10.3389/fpls.2026.1924430)
Supplement: Supplementary file 1 [file SupplementaryFile1.zip › Supplementary Materials/Supplementary Folder 1.zip!/Oidma1_AssemblyScaffolds_Repeatmasked/knownclusterblast/cluster49/jgi.p_Oidma1_60920_mibig_hits.html]

| MIBiG Protein | Description | MIBiG Cluster | MiBiG Product | % ID | % Coverage | BLAST Score | E-value |
| --- | --- | --- | --- | --- | --- | --- | --- |
| AAC01724.1 | putative\_esterase | BGC0000136 | Rifamycin\_biosynthetic\_gene\_cluster | 44 | 49.4669509595 | 201 | 1.6e-51 |
| BAU98033.1 | esterase | BGC0001386 | Isofuranonaphthoquinone\_biosynthetic\_gene\_cluster | 34 | 62.473347548 | 155 | 1.7e-37 |
